# Impact of a Rapid Response Team in Oncological Settings and Associated Outcomes

**DOI:** 10.7759/cureus.115360

**Published:** 2026-08-28

**Authors:** Tauram Javed, Khawaja Muhammad Hamza Zahid, Sadia Sadaqat, Sanaa Afzal, Wajahat Nazir, Ahsun Khan

**Affiliations:** 1 Anesthesiology and Pain Management, Shaukat Khanum Memorial Cancer Hospital and Research Centre, Lahore, PAK

**Keywords:** code status, comfort care only, do-not-resuscitate (dnr) conflicts discussion, do-not-resuscitate (dnr) status, icu patients, rapid response team (rrt), supportive and palliative care

## Abstract

Background and aim

Despite advances in oncological therapies, hospitalized cancer patients remain at high risk of clinical deterioration, morbidity, and mortality. Rapid response teams (RRTs) aim to identify early deterioration and facilitate timely intervention; however, their association with outcomes in oncology populations remains uncertain. This study aimed to describe the characteristics and outcomes associated with RRT activation among hospitalized oncology patients. The primary outcome was hospital mortality; secondary outcomes included ICU transfer, facilitation of do-not-resuscitate (DNR) decisions, and ICU readmission.

Methods

This retrospective, observational, single-center cohort study without a comparison group was conducted over six months (July 1 to December 31, 2019) at Shaukat Khanum Memorial Cancer Hospital and Research Centre, Lahore. Adult oncology inpatients with a National Early Warning Score (NEWS) ≥7 triggering RRT activation were included. Patients with preexisting DNR or comfort-care-only status and non-oncology patients were excluded. Data on demographics, malignancy type, cause of deterioration, interventions, ICU transfer, DNR facilitation, readmissions, and mortality were extracted from the hospital information system and analyzed using IBM SPSS Statistics for Windows, version 27.0 (released 2019; IBM Corp., Armonk, NY, USA).

Results

A total of 302 patients met the inclusion criteria. The most common causes of deterioration were pulmonary (37.2%), infectious (21.3%), and neurological (12.2%) conditions. Following RRT activation, patients were transferred to the ICU in 35.8% of cases, remained on the ward following escalation of care in 30.1%, and had facilitation of a DNR decision in 32.5%. Cardiorespiratory arrest occurred in 1.6% of activations, and ICU readmission occurred in 13.0% of patients transferred to the ICU. The overall hospital mortality rate was 5.4% (54 per 1,000 admissions; 353/6,560), while mortality among patients requiring ICU transfer after RRT activation was substantially higher, at 54.6% (59/108).

Conclusions

RRT activation in this oncology population was associated with ICU transfer, ward-based escalation of care, and facilitation of goals-of-care discussions. Cancer patients who required ICU admission after RRT activation experienced high mortality. As this was a retrospective, single-center, observational study without a comparison group, these findings represent associations rather than evidence of RRT effectiveness; prospective, comparative studies are needed to determine which patients are most likely to benefit from ICU-level versus palliative care.

## Introduction

Hospitalized cancer patients face a disproportionately high risk of acute clinical deterioration compared with the general medical population, driven by disease-related complications, treatment toxicities, and immunocompromised status [1-3]. Despite steady improvements in oncological outcomes over recent decades, the need for timely recognition of and response to deterioration in this population remains a pressing clinical challenge [2]. Rapid response teams (RRTs) have been widely adopted as a hospital-based system for early identification and management of clinically deteriorating patients outside the intensive care unit [4-9]. Understanding how RRTs function in oncology-specific settings is essential to improving care for this vulnerable population.

The literature on RRT effectiveness has yielded mixed results. While multiple single-center studies have reported associations between RRT deployment and reductions in hospital mortality and cardiopulmonary arrest [10], a meta-analysis found only limited evidence to support a significant mortality reduction [11]. Larger multicenter analyses have similarly reported inconsistent associations between RRT implementation and hospital mortality after adjustment for background trends [12]. These inconsistencies may reflect heterogeneity in study populations, RRT activation thresholds, team composition, and the clinical characteristics of patients served. Notably, cancer patients have been underrepresented in many of these studies despite having substantially higher hospital death rates after RRT activation compared with non-cancer patients [13-15]. The specific factors influencing RRT outcomes in oncology populations have not been adequately characterized.

A dedicated evaluation of RRT utilization in a cancer center is therefore needed to understand patterns of deterioration, interventions delivered, and patient outcomes in this population. The primary objective of this study was to describe the outcomes associated with RRT activations in hospitalized cancer patients at a tertiary oncology center, including rates of ICU transfer, do-not-resuscitate (DNR) facilitation, clinical improvement, and mortality.

## Materials and methods

Study design and setting

This retrospective, observational, single-center cohort study was conducted over a period of six months, from July 1, 2019 to December 31, 2019, at Shaukat Khanum Memorial Cancer Hospital and Research Centre, a tertiary care oncology center in Lahore. The study was approved by the Institutional Review Board (IRB) of Shaukat Khanum Memorial Trust (approval EX-07-02-25-01). Data collection commenced after IRB approval.

RRT structure and activation

Before February 2018, initial management of clinically deteriorating patients was performed at the ward level, with escalation to the primary team based on vital-sign abnormalities. Following the establishment of the RRT in February 2018, patients meeting predefined escalation criteria received multidisciplinary assessment and intervention. The RRT team comprised an on-call ICU consultant, an ICU resident, an anesthesia resident, a critical care advanced practice nurse, and a respiratory therapist.

The escalation system followed a two-tier model. Clinical deterioration was initially communicated to the primary treating team (first tier). Patients requiring further assessment or a higher level of care triggered RRT activation (second tier). Following RRT activation, medical interventions were escalated either at the ward level or through ICU transfer; in cases of progressive disease, discussions regarding revision of goals of care were held with the primary team.

Activation criteria for RRTs vary between hospitals worldwide and depend on the severity of clinical deterioration. The primary goals of an RRT are to improve patient outcomes and to facilitate timely decisions regarding goals of care. In this study, RRT activation was triggered by a National Early Warning Score (NEWS) ≥7 [16]. This threshold was selected because it corresponds to the “high-risk” category defined by the Royal College of Physicians, which mandates emergency assessment by a clinical team with critical care competencies [17,18].

Study population

All consecutive in-hospital patients who underwent RRT activation triggered by a NEWS ≥7 during the study period were identified. Patients with a preexisting code status of DNR or comfort-care-only were excluded. Non-oncology patients were also excluded, as the study focus was restricted to the hospitalized cancer population. Over the six-month study period, 302 patients met the inclusion criteria.

Data collection

Data were extracted from the hospital information system (HIS). Whenever a NEWS ≥7 was recorded, an alert was generated to the RRT team via a nursing template in the HIS. The management information system was used to enumerate all patients receiving RRT interventions during the study period. Data collected included demographics, primary malignancy, reason for RRT activation, interventions performed, and patient outcomes following RRT review, including ICU transfer, ICU readmission, and hospital mortality. ICU readmission was defined as any unplanned ICU admission within 48-72 hours of ICU discharge.

Statistical analysis

Data were analyzed using IBM SPSS Statistics for Windows, version 27.0 (released 2019; IBM Corp., Armonk, NY, USA). Normality of continuous variables was assessed using the Kolmogorov-Smirnov test; continuous variables were expressed as median (interquartile range). Categorical data were presented as frequencies and percentages and compared using the chi-square test. Continuous data were compared using the Kruskal-Wallis test.

## Results

Patient characteristics and RRT activation

A total of 302 patients were included, all with a NEWS of ≥7. The RRT team reviewed each patient within 15 minutes of activation and discussed the management plan with the on-call ICU consultant. Table 1 summarizes the distribution of primary malignancies among included patients, and Table 2 shows the frequency distribution of NEWS values in the study cohort; no patients in this cohort had a NEWS of 14. The most common reasons for RRT activation were pulmonary causes (37.2%), infection (21.3%), and neurological causes (12.2%).

**Table 1:** Distribution of primary malignancies among included patients (N = 302).

Primary malignancy	Percentage (%)	Number of patients (n)
Hematological	25.50%	77
Breast cancer	14.80%	45
Urological malignancy	7.40%	22
Lung cancer	7.40%	22
Esophageal cancer	6.40%	19
Head and neck cancer	6.10%	18
Colorectal cancer	6.10%	18
Brain tumor	5.50%	17
Connective tissue malignancy	5.10%	15
Gastric cancer	2.90%	9
Pancreatic cancer	2.90%	9
Gynecological malignancy	2.90%	9
Germ cell tumor	2.20%	7
Cholangiocarcinoma	1.90%	6
Other rare malignancies	2.9%	9

**Table 2:** Frequency distribution of NEWS values in the study cohort (N = 302). No patients in this cohort had a NEWS of 14; the category is retained above for completeness. Percentages are recalculated relative to the total cohort of 302 patients and may not sum to exactly 100% due to rounding. NEWS, National Early Warning Score

NEWS	Number of patients (n)	Percentage (%)
7	35	11.30%
8	62	20%
9	23	7.40%
10	11	3.50%
11	35	11.30%
12	6	1.90%
13	21	6.70%
15	94	30.40%
16	15	4.80%
17	6	1.90%
18	1	0.30%

Interventions and outcomes of RRT activation

This retrospective observational study described the outcomes associated with RRT activations in 302 hospitalized cancer patients at a tertiary oncology center. The principal findings were that approximately one-third of RRT activations resulted in ICU transfer, another third facilitated DNR decision-making, and patients requiring ICU transfer had substantially higher mortality than those managed on the ward. These results suggest that RRTs served a dual role in this oncology setting: acute medical stabilization and facilitation of goals-of-care discussions for patients with progressive disease.

Following RRT activation, patients were transferred to the ICU in 108 cases (35.8%), remained on the ward following escalation of care in 91 cases (30.1%), had facilitation of a DNR decision in 98 cases (32.5%), and experienced cardiorespiratory arrest in five cases (1.6%). These four categories represent mutually exclusive primary triage outcomes and together account for the total cohort (N = 302) (Table 3). Among the 108 patients transferred to the ICU, 14 (13.0%) required ICU readmission within 48-72 hours of ICU discharge.

**Table 3:** Outcomes of RRT interventions. Subgroup quality metric - ICU readmission: 14 patients (13.0%), calculated relative to the 108 patients transferred to the ICU, not the total cohort. Percentages for the primary triage disposition are calculated relative to the total cohort (N = 302). The ICU readmission rate (n = 14) is a subgroup quality metric calculated relative to the total number of patients transferred to the ICU (n = 108). DNR, do-not-resuscitate; RRT, rapid response team

Outcome of RRT intervention	Number of patients (n)	Percentage (%)
ICU transfer	108	35.8%
Prevention of ICU transfer	91	30.1%
Facilitation of DNR decision	98	32.5%
Cardiorespiratory arrest	5	1.60%
Total	302	100%

The overall hospital mortality rate was 5.4%, or 54 per 1,000 admissions, based on 353 deaths among 6,560 total hospital admissions during the study period. Mortality among the subset of patients who required ICU transfer following RRT activation was substantially higher, at 54.6% (59/108), consistent with greater illness severity in this subgroup. As this study lacked a non-RRT control group, this difference should be interpreted as a descriptive association rather than evidence of a causal effect of ICU-level care.

## Discussion

Over the past two decades, RRTs have become increasingly integral to healthcare systems worldwide [13]. Clinical deterioration represents a major global healthcare burden, affecting approximately 3-5% of hospitalized patients. Cancer patients account for approximately 10-15% of all worldwide ICU admissions, with roughly one in seven to one in sixteen cancer patients requiring critical care at some point during their disease trajectory. Although numerous studies have investigated the association between RRTs and hospital mortality rates, the existing body of research has yielded inconclusive results, without a definitive answer regarding the effectiveness of these teams in reducing mortality [13]. This may be due to various factors, including the clinical profile of the study populations and differences in the criteria and thresholds used to activate RRTs.

It is well established that oncology patients are at increased risk of ICU transfer and in-hospital mortality compared with non-cancer patients. Furthermore, major cancer centers have integrated RRTs into their care protocols, complementing advances in chemotherapy and reflecting a trend toward increasingly aggressive treatment approaches [14,15]. In this study, RRT utilization and its associated outcomes were assessed among patients with a NEWS of 7 or higher. The most common cancers among patients requiring RRT review were hematological malignancies, followed by breast cancer and other malignancies.

This observational study found that a substantial proportion of patients with high NEWS showed clinical improvement following RRT intervention and remained on the ward. Common RRT interventions included non-invasive ventilatory support, intravenous fluid resuscitation, broad-spectrum antibiotics, aggressive chest physiotherapy, and other supportive care. RRT activation was also frequently associated with facilitation of DNR decision-making, which may support a patient-centered approach, interdisciplinary collaboration, and adherence to patient wishes near the end of life. Coventry et al. found that RRTs are sometimes called for patients who already have DNR orders; in their study, 15.7% of patients with existing DNR orders still required RRT review, and approximately one-third of RRT calls involved patients receiving end-of-life care [17].

In this study, in-hospital mortality was significantly higher among cancer patients who required ICU transfer after RRT intervention, consistent with previous studies [19]. High mortality rates have similarly been reported among cancer patients following prolonged ICU stays, particularly among those with leukopenia [20]. Danai et al. identified sepsis as one of the major causes of ICU admission among cancer patients and an important contributor to hospital mortality and morbidity, with a higher incidence among patients with hematological malignancy, likely related to associated leukopenia [21]. The reasons for the elevated mortality observed in this subgroup are likely multifactorial and may include advanced cancer stage, comorbidities, treatment-related complications from chemotherapy or radiotherapy, poor performance status, malnutrition, and an immunocompromised state. However, these variables were not captured in this dataset and could not be formally assessed.

These findings are broadly concordant with prior oncology-specific literature. Other studies have similarly reported higher rates of RRT activation among cancer patients compared with general medicine patients, along with significantly higher hospital mortality regardless of ICU transfer status [21]. This consistency suggests that the elevated mortality risk observed in oncology patients requiring RRT and ICU-level care may reflect underlying disease severity rather than differences in the quality of RRT response. Direct comparison across studies, however, remains limited by differences in patient populations, RRT activation criteria, and data completeness.

Because of the retrospective, single-center, observational design of this study and the absence of a comparison group, these findings represent clinical associations rather than direct evidence of causation. It cannot be concluded that the RRT caused the observed avoidance of ICU admission, the facilitation of DNR discussions, or the difference in mortality between groups; these outcomes may instead reflect differences in underlying illness severity, prognosis, or treatment preferences. Prospective or multicenter matched-cohort studies are required to establish the clinical efficacy of RRTs in this population.

This study had several limitations that should be considered when interpreting the results. First, the retrospective, single-center design limits generalizability. Second, the absence of a contemporaneous control group precludes attribution of the observed outcomes to RRT intervention; this study demonstrates associations only, not causation. Third, cancer stage, metastatic status, performance status, and comorbidity data were not uniformly available and could not be adjusted for in the analysis, introducing potential confounding. Fourth, mortality risk factors were not formally explored, owing to these data limitations. Fifth, the sample size was modest, and the study was not powered for subgroup analyses. Finally, as an observational study without a comparison group, the effect of the RRT on patient outcomes cannot be determined.

## Conclusions

This retrospective observational study described the characteristics and outcomes of RRT activations among hospitalized oncology patients. RRT involvement was frequently associated with ICU transfer, ward-based escalation of care, facilitation of DNR discussions, and substantial mortality among patients requiring ICU admission. Given the increasing utilization and expanding role of RRTs in cancer patients, prospective, comparative studies are needed to determine whether RRT interventions improve outcomes in this population and to identify which patients are most likely to benefit from ICU-level versus palliative care.
